# MACF1 Mediates the Impairment of Mechanical Unloading on Osteoblast Differentiation via F-Actin/ERK/Runx2 Axis

**DOI:** 10.3390/cells15161448

**Published:** 2026-08-11

**Authors:** Lifang Hu, Kang Ru, Wenjin Zhong, Linlin Wang, Zizhan Huang, Lei Qiao, Zhihao Chen, Airong Qian

**Affiliations:** Lab for Bone Metabolism, Xi’an Key Laboratory of Special Medicine and Health Engineering, Key Lab for Space Biosciences and Biotechnology, Research Center for Special Medicine and Health Systems Engineering, School of Life Science and Technology, Northwestern Polytechnical University, Xi’an 710072, China; hulifang@nwpu.edu.cn (L.H.); rukang@mail.nwpu.edu.cn (K.R.); zhongwenjin@mail.nwpu.edu.cn (W.Z.); wllin@mail.nwpu.edu.cn (L.W.); qiaol@mail.nwpu.edu.cn (L.Q.); chenzhihao@nwpu.edu.cn (Z.C.)

**Keywords:** MACF1, mechanical unloading, osteoblast differentiation, F-actin, ERK, Runx2

## Abstract

**Highlights:**

- MACF1 is essential for osteoblasts’ response to mechanical unloading.
- MACF1 functions as a mechanotransduction mediator and mediates the inhibitory effect of mechanical unloading on osteoblast differentiation via the F-actin/ERK/Runx2 axis.

**What are the main findings?**

- This study reveals, for the first time, the mechanotransduction role of MACF1 in mechanical unloading whilst suppressing osteoblast differentiation.
- MACF1 mediates the inhibitory effect of mechanical unloading on osteoblast differentiation via the F-actin/ERK/Runx2 axis.

**What are the implications of the main findings?**

- The necessity of MACF1 for osteoblasts’ response to mechanical stimuli.
- A novel insight of mechanical stimuli regulating osteoblast differentiation and cell mechanotransduction.

**Abstract:**

Decreased osteoblast differentiation contributes to bone loss induced by mechanical unloading. However, the underlying mechanism is still unclear. We previously found that microtubule actin crosslinking factor 1 (MACF1), a key cytoskeletal protein, plays an important role in regulating osteoblast differentiation, while the role of MACF1 in mechanical unloading suppressing osteoblast differentiation remains unclear. Here, an MACF1-knockdown (MACF1-KD) osteoblast cell line and primary osteoblasts were subjected to mechanical unloading conducted by a random positioning machine (RPM). Osteoblast differentiation was evaluated by alkaline phosphatase (ALP) staining and real-time PCR. F-actin distribution was examined by immunofluorescence staining. Western blot was adopted to detect the protein levels. Moreover, cytochalasin B and PD98059 were applied to disrupt F-actin and inhibit extracellular signal-regulated kinase (ERK) activity, respectively, to confirm the mechanism. The results show that MACF1 is significantly downregulated in osteoblasts by mechanical unloading together with decreased osteoblast differentiation. MACF1-KD osteoblasts exhibit reduced differentiation capacity and are insensitive to mechanical unloading. Mechanistically, MACF1 mediates the suppression of mechanical unloading on osteoblast differentiation by regulating F-actin distribution and the downstream ERK/Runx2 signaling. Furthermore, F-actin disruption and ERK inhibition assays confirm that MACF1 mediates the impairment of mechanical unloading on osteoblast differentiation via the F-actin/ERK/Runx2 axis. In conclusion, this study reveals MACF1 as a mechanotransduction mediator for mechanical unloading, inhibiting osteoblast differentiation via F-actin/ERK/Runx2, and contributes to a novel mechanistic insight of cell mechanotransduction.

## 1. Introduction

Mechanical stimuli play a vital role in regulating osteoblast differentiation, bone formation and bone homeostasis [1,2,3,4]. Mechanical unloading (MU) due to long-term spaceflight, prolonged bed rest, and immobilization causes bone loss, which is a key concern of human spaceflight and public health [5,6,7,8]. Osteoblasts, bone-forming cells, sense and respond to mechanical stimuli to participate in bone homeostasis. Accumulating evidence has demonstrated that decreased osteoblast differentiation mainly contributes to MU-indued bone loss [9,10,11]. Colucci et al. reported that MU due to spaceflight suppresses the expression of key osteogenic genes runt-related transcription factor 2 (Runx2) and Osterix [12]. Shi et al. showed that MU conducted by a 2D clinostat dramatically reduced alkaline phosphatase (ALP) activity and inhibited osteoblast differentiation [13]. Other recent studies also show decreased osteoblast differentiation and reduced bone formation, which lead to bone loss under MU conditions [14,15]. All of this demonstrates the crucial contribution of decreased osteoblast differentiation to MU-indued bone loss. However, the underlying mechanism affecting osteoblast differentiation under MU is still unclear.

Microtubule actin crosslinking factor 1 (MACF1), a key cytoskeletal protein belonging to the spectraplakin family, is essential in regulating F-actin and microtubule dynamics [16,17,18]. MACF1 shows versatile roles in regulating cell migration, differentiation and many biological processes [19,20,21,22]. We have previously found that MACF1 plays an important role in regulating the distribution of F-actin and microtubules [23]. Moreover, we have found that knockdown of MACF1 in osteoblasts suppresses osteoblast differentiation [24,25], while overexpression of MACF1 promotes osteoblast differentiation [26]. Further, conditional knockout of MACF1 in osteoblast lineage cells in mice significantly inhibits osteoblast differentiation and bone formation [27]. All these findings demonstrate the importance of MACF1 in regulating osteoblast differentiation. However, whether MACF1 participates in the decreased osteoblast differentiation induced by MU is unknown.

In the current study, we investigated the role of MACF1 in MU inhibiting osteoblast differentiation and the possible underlying mechanism. We found that MU inhibits osteoblast differentiation and concurrently downregulates MACF1 expression, while MACF1-knockdown (MACF1-KD) osteoblasts showed decreased osteoblast differentiation and did not respond to MU conditions. In addition, we revealed that MACF1 regulated the arrangement of F-actin, which is critical for osteoblast differentiation. We further demonstrated that ERK activation, which is regulated by F-actin, was inhibited by MU and MACF1-KD, and ERK inactivation suppressed Runx2 expression and osteoblast differentiation. These findings demonstrate that MACF1 acts as a mechanotransduction mediator to mediate MU inhibiting osteoblast differentiation via F-actin/ERK/Runx2 signal axis.

Collectively, our findings extend our understanding of cell mechanotransduction and cytoskeletal protein, providing novel insight for the regulation of MU in osteoblast differentiation.

## 2. Materials and Methods

### 2.1. Cell Culture

The murine MC3T3-E1 osteoblastic cell line was kindly provided by Dr. Hong Zhou at the University of Sydney. A stable MACF1-KD MC3T3-E1 cell line was established using highly specific lentivirus-mediated shRNA (short hairpin RNA), which has been screened out from four independent MACF1-targeted shRNAs based on the knockdown efficiency and the resulting effects on osteoblast differentiation [24]. The MACF1-KD cell line serves as a well-validated model in our laboratory [23,24,25]. All cell lines were confirmed to be free of mycoplasma and other microbial contamination. The cells were cultured in alpha-Minimum Essential Medium (α-MEM, Gibco, 11900–024, Carlsbad, CA, USA) containing 10% fetal bovine serum (FBS; Biological Industries, 04-001-1A, Kibbutz Beit Haemek, Israel), 10 mM L-glutamine (Sigma, G8540, St Louis, MO, USA), 1% penicillin (Amresco, 0242, Solon, OH, USA) and 1% streptomycin (Amresco, 0382, Solon, OH, USA). Cell cultures were maintained in a humidified, 37 °C, 5% CO_2_ incubator (Thermo Fisher Scientific, Waltham, MA, USA). For the differentiation assay, cells were allowed to grow to confluence, and the medium was changed to osteogenic medium (supplemented with 50 μg/mL ascorbic acid and 10 mM β-GP (Sigma-Aldrich, St Louis, MO, USA)). For cytochalasin B (Cyto B) experiments, cells were treated with 1 μM Cyto B for 24 h (hours). For the experiments with PD98059, the inhibitor of ERK activation, cells were treated with 20 μM PD98059 for 48 h.

### 2.2. Mechanical Unloading (MU) Condition

A desktop random positioning machine (RPM, the Center for Space Science and Applied Research of Chinese Academy of Sciences) was used to enact the MU condition, as previously described [28,29].

As our previous studies reported [28], for MU studies, osteoblasts were seeded on coverslips at a density of 8 × 10^4^ cells per well in 6-well plates and cultured with α-MEM medium containing 10% FBS, 100 μg/mL streptomycin and 100 units/mL penicillin at 37 °C and 5% CO_2_. When the cells became confluent after around 2 days, the cells were placed into cell culture flasks. To minimize the confounding effects of fluid shear stress, all the cell culture flasks were completely filled with osteogenic media to exclude any air bubbles and fixed into the culture vessel of the RPM. The RPM was rotated randomly in the range of 0–10 rpm for 24 h and 48 h respectively. The cells of the corresponding control group were seeded and handled the same as the MU group and were placed in the same incubator without rotation.

### 2.3. Alkaline Phosphatase (ALP) Staining

The ALP of osteoblasts was stained by using BCIP (5-bromo-4-chloro-3-indolyl phosphate)/NBT (nitro blue tetrazolium) ALP Color Development Kit (Beyotime Biotechnology, Shanghai, China) according to the manufacturer’s instruction, as previously described [25]. Briefly, cells were fixed in 10% buffered formaldehyde for 20 min, followed by washing with phosphate-buffered saline (PBS, pH7.4) three times, 5 min/time, and then were stained using BCIP/NBT solution. The staining reaction was stopped by washing with distilled water and the cell staining was imaged by a scanner (CanoScan 9000 F Mark II, Canon, Bangkok, Thailand).

### 2.4. Real-Time PCR

Total RNA was extracted from cells using Total RNA Kit II (Omega, Norcross, GA, USA, R6934-02) according to the manufacturer’s instruction; 1 µg of total RNA sample was reverse-transcribed into complementary DNA (cDNA) using a PrimeScript™ RT reagent kit (Omega Bio-Tek, RR037A). The expression of genes was detected by real-time PCR carried out in a C1000™ Thermal Cycler (Bio-Rad Labs, Hercules, CA, USA) with specific primers (Table 1) and a ChamQTM SYBR^®^ qPCR Master Mix Kit (Vazyme, Nanjing, China, Q311-02/03). Samples were amplified using the following program: pre-denaturation at 95 °C for 30 s, followed by 45 cycles of 95 °C for 10 s, 58 °C or 60 °C for 30 s, and 72 °C for 5 s. Fluorescence detection was performed at the annealing phase at 80 °C to exclude the interference of primer dimers. The relative expression of genes was calculated via the 2^−ΔΔCt^ method [30] by using glyceraldehyde-3-phosphate dehydrogenase (GAPDH) for normalization of the threshold cycle (Ct) value.

### 2.5. Western Blot

Western blot was adopted to detect the protein level. Cellular total protein extracts were prepared on ice using RIPA (Solarbio, Beijing, China) supplemented with 1% PMSF (Solarbio, Beijing, China). The protein concentration was determined by using BCA protein assay kit (Solarbio, Beijing, China) and an equal amount of proteins for each sample was subjected to 10% SDS-PAGE and transblotted to a nitrocellulose membrane (Millipore Corporation, MA, USA). After incubation with the blocking buffer (5% nonfat milk or 5% BSA), the membrane was incubated with appropriate primary antibodies against the following proteins in accordance with the manufacturer’s instructions at 4 °C overnight: MACF1 (Proteintech, Wuhan, China), ERK, p-ERK (Thr202/Tyr204), Runx2, p-Runx2 (Ser-340) (Affinity Biosciences, Changzhou, China) and GAPDH (Proteintech, Wuhan, China). The horseradish peroxidase (HRP) conjugated secondary antibody (Proteintech, Wuhan, China) was further used. Protein bands were visualized by chemiluminescence using an ECL kit (Proteintech, Wuhan, China) and exposed to ChemiDoc XRS to detect the Western bands. Protein band intensities were quantified using ImageJ software 1.54t version (National Institutes of Health, NIH). GAPDH was used as the internal control.

### 2.6. Fluorescence Staining

Fluorescence staining of F-actin was conducted by using Actin-Stain 555 Phalloidin (Cytoskeleton, Denver, CO, USA) according to the manufacturer’s instructions. Briefly, cells were fixed with 4% paraformaldehyde for 20 min and rinsed for 5 min with TBS, 10 min with TBS-0.5% TritonX-100 (TX), and 3 × 5 min with TBS-0.1%TX, and then blocked with 2% bovine serum albumin (BSA) and 0.1% sodium azide in 0.1% TX (TBS) for 10 min at room temperature. Then, the cells were incubated with Phalloidin at 4 °C overnight. After overnight incubation, the cells were incubated with 5 μg/mL DAPI (4′,6-diamidino-2-fenilindol) (Beyotime, Shanghai, China) for 10 min to stain the nucleus. Finally, the cells were enveloped with Fluoromount-G (Southern Biotech, Birmingham, AL, USA) and observed by laser scanning confocal microscopy (Leica TCS SP5, Wetzlar, Germany).

F-actin was quantified using Image J software (National Institutes of Health, Bethesda, MD, USA). Briefly, the F-actin network was skeletonized for morphometric analysis. The average F-actin branch length was calculated to assess filament continuity, and the F-actin fragmentation index was used to evaluate the degree of actin filament disruption. Decreased average branch length, combined with an elevated fragmentation index, was interpreted as increased F-actin disorganization and fragmentation.

### 2.7. Statistical Analysis

All experiments were independently performed three times. The data were analyzed using Graphpad Prism 7 software and all results were presented as mean value ± standard deviation (SD). Student’s *t*-test was used to conduct comparisons between two groups and the two-way ANOVA with post hoc Tukey’s test was adopted for multiple group comparisons. Statistical significance was set at *p* < 0.05 (* *p* < 0.05; ** *p* < 0.01; *** *p* < 0.001).

## 3. Results

### 3.1. MU Inhibits Osteoblast Differentiation and Concurrently Downregulates MACF1 Expression

**Figure 1:**
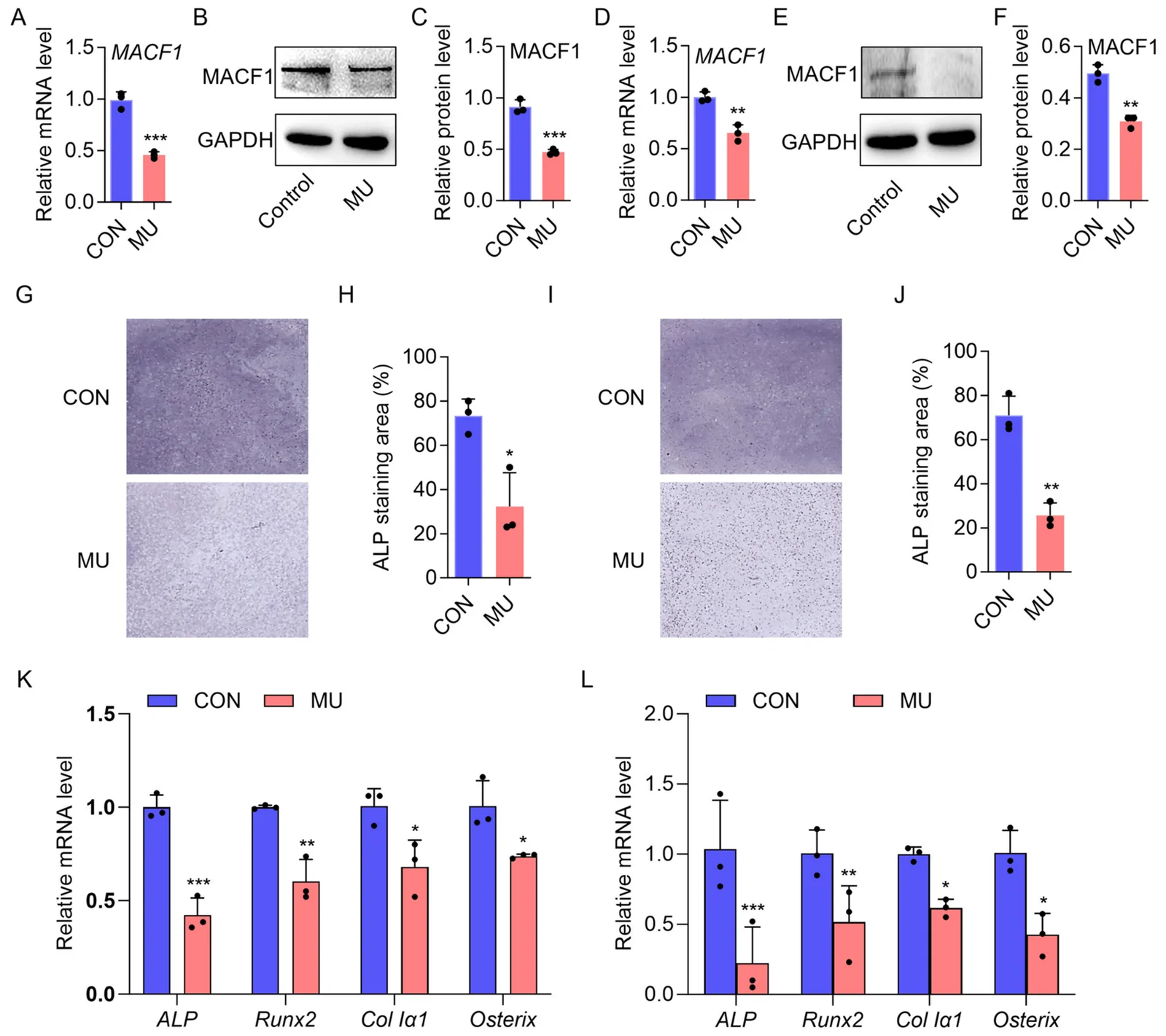
Mechanical unloading (MU) inhibits MACF1 expression and osteoblast differentiation. (**A**) The expression of MACF1 in MC3T3-E1 osteoblasts was examined by real-time PCR after 24 h treatment of MU. (**B**,**C**) Representative Western blots of MACF1 in MC3T3-E1 cells treated by 24 h MU (**B**) and the quantification results with GAPDH as internal control (**C**). (**D**) The expression of MACF1 in MC3T3-E1 cells was examined by real-time PCR after 48 h treatment of MU. (**E**,**F**) Representative Western blots of MACF1 in MC3T3-E1 cells treated by 48 h MU (**E**) and the quantification results with GAPDH as internal control (**F**). (**G**,**H**) ALP activity of MC3T3-E1 cells was detected by ALP staining after 24 h treatment of MU (**G**) and the quantification of ALP staining area (**H**). (**I**,**J**) ALP activity of MC3T3-E1 cells was detected by ALP staining after 48 h treatment of MU (**I**) and the quantification of ALP staining area (**J**). (**K**,**L**) The expression of ALP, Runx2, ColIα1 and Osterix in MC3T3-E1 cells examined by real-time PCR after 24 h (**K**) and 48 h (**L**) treatment of MU respectively. CON: Normal control condition, MU: mechanical unloading. Data are presented as mean value ± SD and Student’s *t*-test was used for statistical analysis. * *p* < 0.05, ** *p* < 0.01, *** *p* < 0.001.

Decreased bone formation due to reduced osteoblast differentiation is one main cause of bone loss induced by MU. However, the underlying mechanism is still unclear. Our previous findings show that MACF1 is mechano-responsive and promotes osteoblast differentiation and bone formation [24,25,26]. However, whether MACF1 plays roles in MU suppressing osteoblast differentiation is unknown. Here, we showed that MU conducted by RPM significantly decreased MACF1 mRNA and protein expression after 24 h (Figure 1A–C) and 48 h treatments (Figure 1D–F) in osteoblast cell line MC3T3-E1. Meanwhile, osteoblast differentiation was inhibited by MU, presenting as decreased ALP activity (Figure 1G–J) and reduced expression of osteogenic genes ALP, Runx2, Osterix, and type I collagen α1 (ColIα1) (Figure 1K,L) in MC3T3-E1 cells. Moreover, the same findings were obtained in primary osteoblasts under MU conditions (Figure S1), showing as decreased MACF1 mRNA and protein levels after 24 h (Figure S1A–C) and 48 h treatments (Figure S1D–F), and reduced ALP activity (Figure S1G–J) and expression of osteogenic genes *ALP*, *Runx2*, *Osterix*, and *ColIα1* (Figure S1K,L). As our previous studies have demonstrated that a low level of MACF1 inhibits osteoblast differentiation [24,25], these results indicate the involvement of MACF1 in MU inhibiting osteoblast differentiation.

### 3.2. MACF1 Mediates the Inhibition Effect of MU on Osteoblast Differentiation

**Figure 2:**
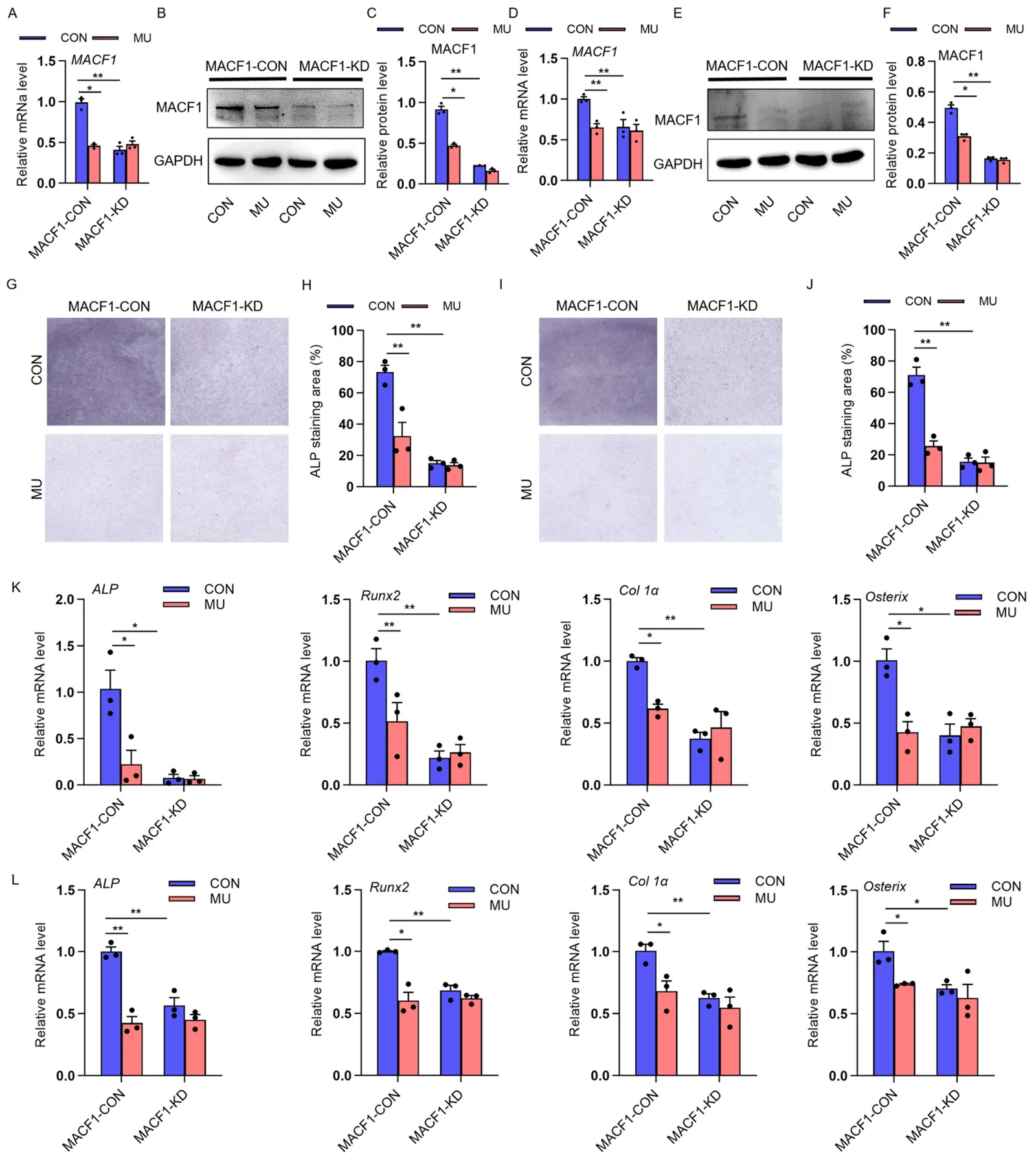
MACF1 knockdown inhibits osteoblast differentiation and is involved in the inhibition effect of MU on osteoblast differentiation. (**A**) The expression of MACF1 examined by real-time PCR after 24 h treatment of MU in control (MACF1-CON) and MACF1-KD cells. (**B**,**C**) Representative Western blots of MACF1 treated by 24 h MU in MACF1-CON and MACF1-KD cells (**B**) and the quantification results with GAPDH as internal control (**C**). (**D**) The expression of MACF1 examined by real-time PCR after 48 h treatment of MU in MACF1-CON and MACF1-KD cells. (**E**,**F**) Representative Western blots of MACF1 treated by 48 h MU in MACF1-CON and MACF1-KD cells (**E**) and the quantification results with GAPDH as internal control (**F**). (**G**,**H**) ALP activity was detected by ALP staining after 24 h treatment of MU in MACF1-CON and MACF1-KD cells (**G**) and the quantification of ALP staining area (**H**). (**I**,**J**) ALP activity was detected by ALP staining after 48 h treatment of MU in MACF1-CON and MACF1-KD cells (**I**) and the quantification of ALP staining area (**J**). (**K**,**L**) The expression of ALP, runx2, ColIα1 and Osterix examined by real-time PCR after 24 h (**K**) and 48 h (**L**) treatment of MU in MACF1-CON and MACF1-KD cells, respectively. CON: Normal control condition, MU: mechanical unloading, MACF1-CON: control osteoblasts, MACF1-KD: MACF1-knockdown cells. Data are presented as mean value ± SD and the two-way ANOVA with post hoc Tukey’s test was used for statistical analysis. * *p* < 0.05, ** *p* < 0.01.

As our previous study shows that a low level of MACF1 inhibits osteoblast differentiation [25], the above results suggest that MACF1 may play an important role in MU inhibiting osteoblast differentiation. To determine the role of MACF1 in MU suppressing osteoblast differentiation, the stable MACF1-KD MC3T3-E1 osteoblast cell line and the corresponding control cells (MACF1-CON) were adopted for further study. MACF1-KD osteoblasts and MACF1-CON cells were cultured with osteogenic medium under MU conditions (MU). We found that in MACF1-CON cells, MACF1 expression was significantly decreased by 24 h and 48 h treatment of MU (Figure 2A–F). Moreover, similar results were obtained for osteoblast differentiation ability. The ALP activity was dramatically reduced in the MACF1-CON cells treated by MU (Figure 2G–J). In addition, the expression of osteogenic genes *ALP*, *Runx2*, *Osterix*, and *Col Iα1* was significantly downregulated in MACF1-CON cells treated by MU (Figure 2K,L). Interestingly, in MACF1-KD cells, the ALP activity and the expression of osteogenic marker genes were reduced to a level equivalent to that observed in MACF1-CON cells by MU, and MU did not cause a further decrease (Figure 2G–L). Moreover, the same findings were found in primary osteoblasts. The siRNA of MACF1 was used to knock down MACF1 expression in primary osteoblasts, and both the control cells (MACF1-CON) and MACF1-knockdown (si-MACF1) cells were treated by MU. The 24 h and 48 h treatment of MU dramatically reduced MACF1 expression in MACF1-CON primary osteoblasts (Figure S2A–F). In addition, MU treatment significantly suppressed ALP activity and the expression of osteogenic genes *ALP*, *Runx2*, *Col Iα1*, and *Osterix* (Figure S2G–L). In si-MACF1 cells, the ALP activity and the expression of osteogenic marker genes were reduced to a level equivalent to that observed in MACF1-CON cells by MU, and MU did not cause a further decrease (Figure S2G–L). All these findings reveal the critical role of MACF1 in MU suppressing osteoblast differentiation and indicate its necessity for osteoblasts responding to MU conditions.

### 3.3. MACF1 Mediates the Inhibition Effect of MU on Osteoblast Differentiation by Regulating F-Actin Distribution

**Figure 3:**
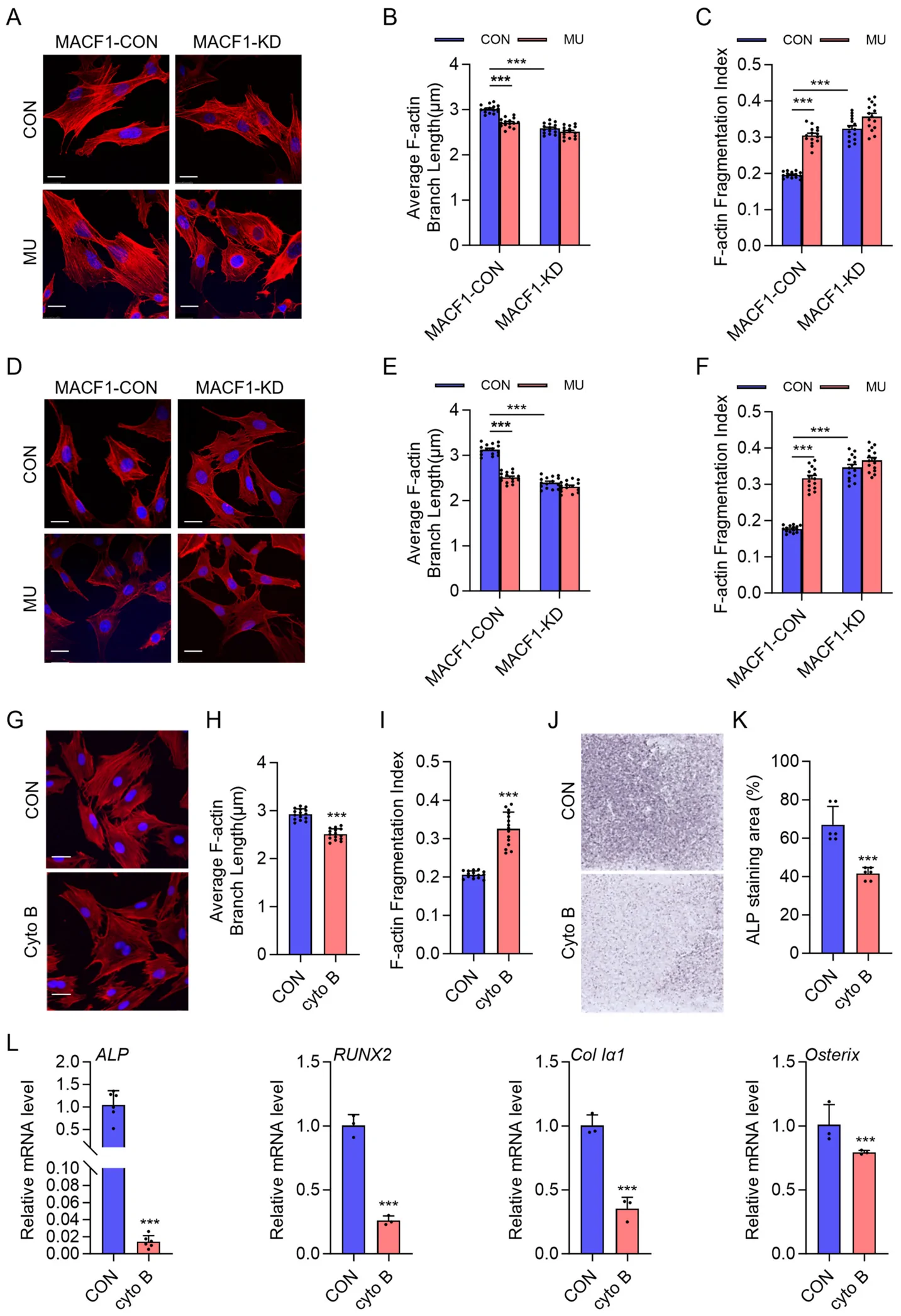
MACF1 knockdown alters F-actin distribution and MACF1 mediates the inhibition effect of MU on osteoblast differentiation by regulating F-actin distribution. (**A**) F-actin distribution was detected by fluorescence staining after 24 h treatment of MU in MACF1-CON and MACF1-KD cells. Bar: 50 μm. (**B**,**C**) Quantitative analysis of average F-actin branch length (**B**) and the F-actin fragmentation index (**C**) after 24 h of MU treatment in MACF1-CON and MACF1-KD cells. (**D**) F-actin distribution was detected by fluorescence staining after 48 h treatment of MU in MACF1-CON and MACF1-KD cells. Bar: 50 μm. (**E**,**F**) Quantitative analysis of average F-actin branch length (**E**) and the F-actin fragmentation index (**F**) after 48 h of MU treatment in MACF1-CON and MACF1-KD cells. (**G**) F-actin distribution in MC3T3-E1 cells was detected by fluorescence staining after 1μM Cyto B treatment for 24 h. Bar: 50 μm. (**H**,**I**) Quantitative analysis of average F-actin branch length and the F-actin fragmentation index after 1μM Cyto B treatment for 24 h. (**J**,**K**) ALP activity was detected by ALP staining after 1μM Cyto B treatment for 24 h (**J**) and the quantification of ALP staining area (**K**). (**L**) The expression of ALP, Runx2, ColIα1 and Osterix examined by real-time PCR after 1 μM Cyto B treatment for 24 h. CON: Normal control condition, MU: mechanical unloading, MACF1-CON: control osteoblasts, MACF1-KD: MACF1-knockdown cells, Cyto B: cytochalasin B treatment. Data are presented as mean value ± SD and Student’s *t*-test was used for statistical analysis. *** *p* < 0.001.

MACF1 is a key regulator of the dynamics of F-actin cytoskeleton, which is mechanosensitive and plays roles in regulating osteoblast differentiation [31,32,33]. Thus, we speculated that MACF1 may regulate osteoblast differentiation via F-actin under MU conditions. To test this hypothesis, the effects of MU on F-actin distribution in both control cells and MACF1-KD cells were detected by fluorescence staining. The results showed that compared to the well-organized and distributed F-actin in control cells (MACF1-CON) of the control group, MU induced redistribution of F-actin in MACF1-CON cells, showing the localization mainly at the cell periphery (Figure 3A,D). However, in MACF1-KD cells either treated or nor with MU, the F-actin distribution was similar to the MACF1-CON cells treated by MU (Figure 3A,D). The quantitative analysis showed that MU significantly reduced the average F-actin branch length and increased the F-actin fragmentation index in MACF1-CON osteoblasts (Figure 3B,C,E,F), indicating the disruption and fragmentation of the F-actin. MACF1-KD cells exhibited a similarly shortened F-actin branch length and elevated fragmentation index under control conditions compared to MACF1-CON cells treated by MU, and MU did not cause further alterations in MACF1-KD cells (Figure 3B,C,E,F). These findings demonstrate that MU and MACF1-KD are associated with impaired F-actin continuity, increased filament fragmentation, and altered cytoskeletal organization. In addition, these findings were confirmed in primary osteoblasts (Figure S3A–F). The results demonstrate the regulatory role of MU on F-actin distribution and indicate that MACF1 mediates the regulatory effect of MU on F-actin distribution in osteoblasts.

Further, to verify the role of F-actin in osteoblast differentiation, Cyto B was adopted to interrupt the F-actin distribution to detect the changes in osteoblast differentiation ability in both MC3T3-E1 osteoblasts and primary osteoblasts. We found that Cyto B induced similar changes in F-actin distribution to control cells treated by MU and MACF1-KD, presenting as the main distribution of F-actin at the cell periphery with decreased F-actin branch length and increased fragmentation index (Figure 3G–I and Figure S3G–I). In addition, the ALP staining showed that the ALP activity was significantly reduced by Cyto B treatment (Figure 3J,K and Figure S3J,K). Moreover, the expression of osteogenic genes *ALP*, *Runx2*, *Osterix*, and *Col Iα1* was downregulated by Cyto B treatment (Figure 3L and Figure S3L). All these findings demonstrate that MACF1-regulated F-actin remodeling is a critically involved pathway in mediating the inhibition effect of MU on osteoblast differentiation.

### 3.4. ERK Signaling Participates in the Meditation of MACF1 on MU Inhibiting Osteoblast Differentiation

**Figure 4:**
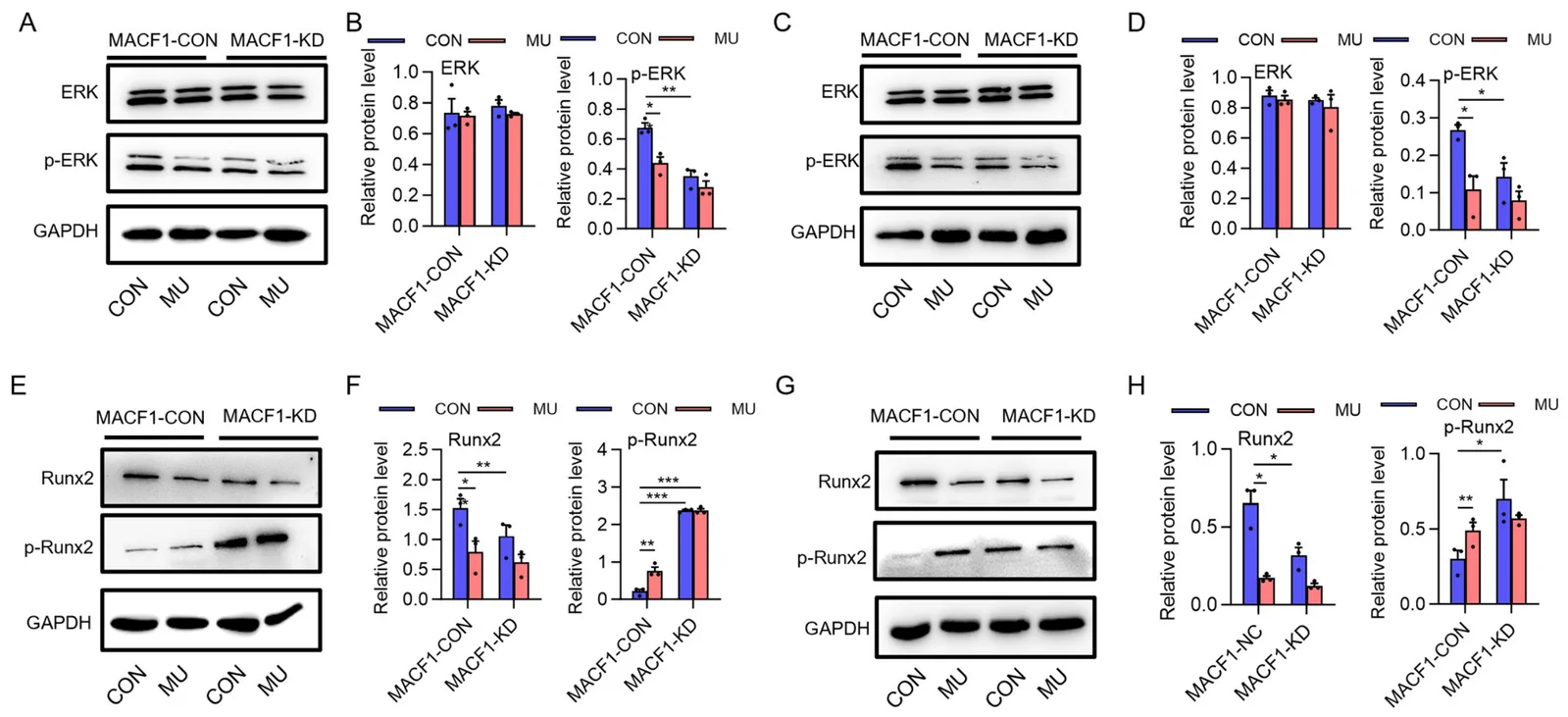
ERK signaling is inhibited by MU and is involved in the meditation of MACF1 on MU inhibiting osteoblast differentiation. (**A**,**B**) Representative Western blots of ERK and p-ERK treated by 24 h MU in MACF1-CON and MACF1-KD cells (**A**) and the quantification results with GAPDH as internal control (**B**). (**C**,**D**) Representative Western blots of ERK and p-ERK treated by 48 h MU in MACF1-CON and MACF1-KD cells (**C**) and the quantification results with GAPDH as internal control (**D**). (**E**,**F**) Representative Western blots of Runx2 and p-Runx2 treated by 24 h MU in MACF1-CON and MACF1-KD cells (**E**) and the quantification results with GAPDH as internal control (**F**). (**G**,**H**) Representative Western blots of Runx2 and p-Runx2 treated by 48 h MU in MACF1-CON and MACF1-KD cells (**G**) and the quantification results with GAPDH as internal control (**H**). CON: Normal control condition, MU: mechanical unloading, MACF1-CON: control osteoblasts, MACF1-KD: MACF1-knockdown cells. Data are presented as mean value ± SD and the two-way ANOVA with post hoc Tukey’s test was used for statistical analysis. * *p* < 0.05, ** *p* < 0.01, *** *p* < 0.001.

As studies have demonstrated that F-actin plays a key role in regulating ERK signaling, which is important for osteoblast differentiation by regulating Runx2, the key transcription factor for osteoblast differentiation [31,32], we hypothesized that ERK signaling may participate in the meditation of MACF1 on MU inhibiting osteoblast differentiation. Thus, the effects of MU on ERK signaling were detected in both control and MACF1-KD osteoblasts. The results showed that after 24 h and 48 h treatment of MU, the phosphorylated ERK (p-ERK) level was significantly reduced in control cells, while there was no significant alteration of p-ERK level between MACF1-KD cells and MACF1-KD cells treated by MU (Figure 4A–D). Further, the effects of MU on the expression and phosphorylation of key osteogenic transcription factor Runx2, downstream of ERK signaling, was detected in both control and MACF1-KD cells. As shown in Figure 4E–H, MU significantly inhibited the total protein level of Runx2 but increased the p-Runx2 (Ser340) in control cells. MACF1-KD also reduced the total Runx2 but increased the p-Runx2 level compared to the control group (Figure 4E–H). However, MU treatment did not cause further changes in MACF1-KD cells (Figure 4E–H). In classical genetic and pathway analysis, this lack of an additive effect in the knockdown (KD) background provides strong evidence of epistasis. If MU bypassed MACF1 to inhibit ERK phosphorylation via an alternative pathway, we would expect a further decrease in ERK phosphorylation when MU was added to MACF1-KD cells. Because MU has no effect on ERK phosphorylation when MACF1 is missing, it logically suggests that the inhibitory effect of MU on ERK is dependent on the downregulation of MACF1. Thus, these findings provide mechanistic proof of the MU/MACF1/ERK axis.

### 3.5. MACF1 Mediates the Inhibition Effect of MU on Osteoblast Differentiation Through F-Actin/ERK/Runx2 Signal Axis

**Figure 5:**
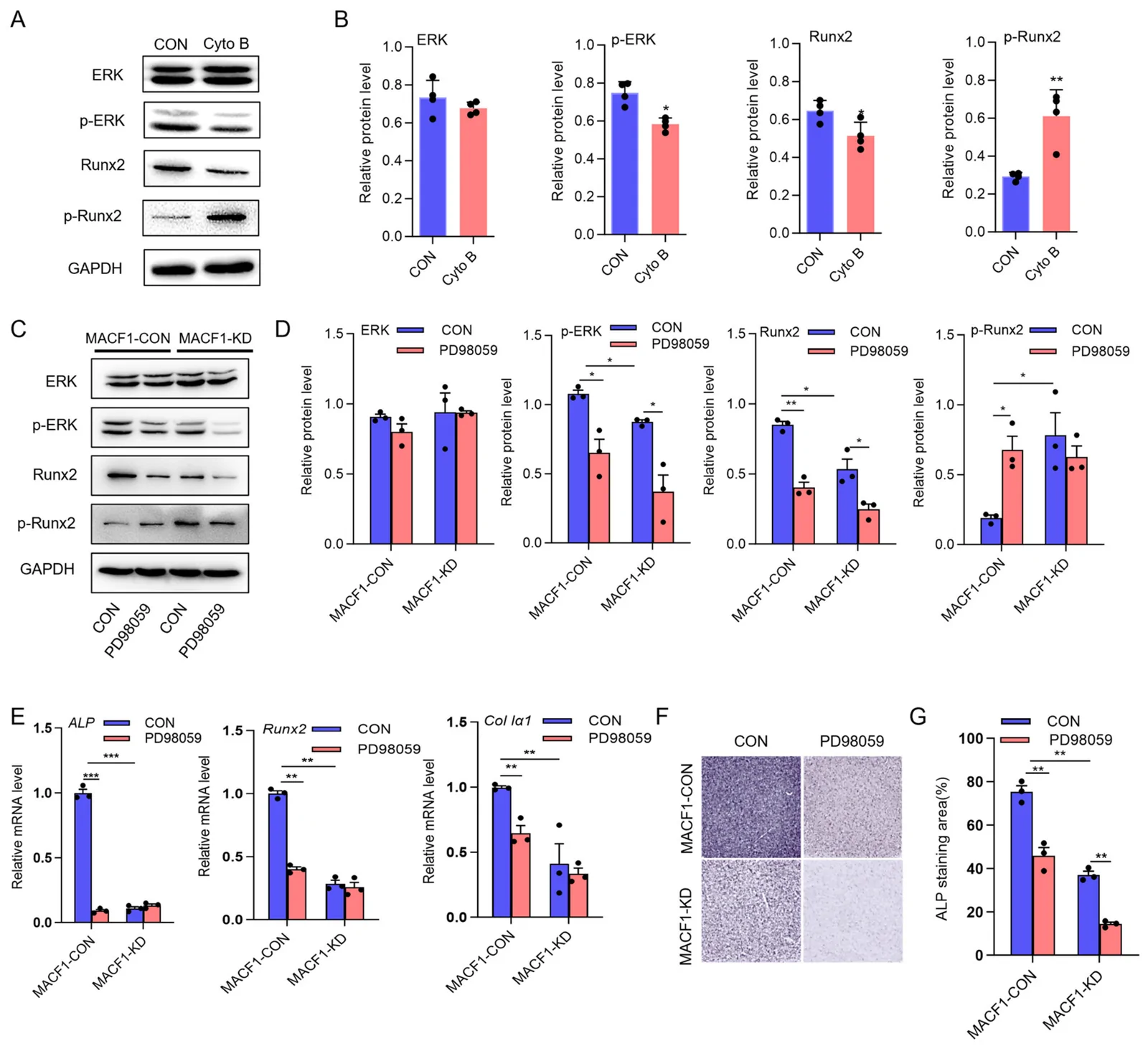
F-actin/ERK/Runx2 signal axis participates in the mediation of MACF1 on MU inhibiting osteoblast differentiation. (**A**,**B**) Representative Western blots of ERK, p-ERK, Runx2, and p-Runx2 of MC3T3-E1 cells treated by Cyto B for 24 h (**A**) and the quantification results with GAPDH as internal control (**B**). (**C**,**D**) Representative Western blots of ERK, p-ERK, Runx2, and p-Runx2 in MACF1-CON and MACF1-KD cells treated by PD98059 for 48 h (**C**) and the quantification results with GAPDH as internal control (**D**). (**E**) The expression of *ALP*, *Runx2*, and *ColIα1* was examined by real-time PCR after PD98059 treatment for 48 h in MACF1-CON and MACF1-KD cells. (**F**,**G**) ALP activity was detected by *ALP* staining after PD98059 treatment for 48 h in MACF1-CON and MACF1-KD cells (**F**) and the quantification of *ALP* staining area (**G**). CON: Normal control condition, Cyto B: cytochalasin B treatment, MACF1-CON: control osteoblasts, MACF1-KD: MACF1-knockdown cells, PD98059: PD98059 treatment. Data are presented as mean value ± SD. Student’s *t*-test was used for two groups comparation and two-way ANOVA with post hoc Tukey’s test was used for multiple group comparation. * *p* < 0.05, ** *p* < 0.01, *** *p* < 0.001.

As MACF1 regulates F-actin distribution and ERK activation, we investigated whether ERK functions downstream of F-actin. The effect of F-actin on ERK signaling was detected by adopting Cyto B treatment. As shown in Figure 5A,B, Cyto B treatment significantly inhibited p-ERK, an indicator for activation of ERK signaling. In addition, the expression of Runx2 was decreased by Cyto B treatment, while p-Runx2 was increased (Figure 5A,B). These results reveal that F-actin regulates the ERK/Runx2 signal axis.

The above findings suggest that MACF1 may mediate the inhibition effect of MU on osteoblast differentiation by regulating F-actin distribution to affect ERK/Runx2 signaling. To confirm the role of ERK in regulating osteoblast differentiation, we treated both MACF1-CON and MACF1-KD cells with PD98059, which is the inhibitor of ERK activation. The results showed that PD98059 significantly decreased p-ERK level in both MACF1-CON cells and MACF1-KD cells (Figure 5C,D). Together with the observation that MACF1-KD alone reduced p-ERK levels (Figure 4A–D), these results indicate that MACF1 is required for ERK activation in osteoblasts and suggest that ERK may act downstream of MACF1. In addition, total Runx2 protein level was significantly decreased by PD98059 in both MACF1-CON and MACF1-KD cells (Figure 5C,D) but the p-Runx2 was dramatically increased by PD98059 in MACF1-CON cells. These findings are similar to the effects of MU treatment. Further, we found that PD98059 treatment dramatically downregulated the expression of osteogenic genes *ALP*, *ColIα1* and *Runx2* in MACF1-CON cells (Figure 5E). Moreover, ALP staining showed that PD98059 treatment significantly inhibited the *ALP* activity in both MACF1-CON cells and MACF1-KD cells (Figure 5F,G). These findings show that ERK activation promotes osteoblast differentiation and confirm the involvement of MACF1 in mediating the inhibition effect of MU on osteoblast differentiation via the F-actin/ERK/Runx2 signal axis.

## 4. Discussion

Mechanical stimuli play important roles in regulating osteoblast differentiation to maintain bone homeostasis [3,4]. Osteoblasts respond to MU and show reduced differentiation ability [9,29]. However, the underlying mechanism is still unclear. The present study shows that MU inhibits osteoblast differentiation and concurrently downregulates the expression of MACF1. Moreover, knockdown of MACF1 in both MC3T3-E1 osteoblasts and primary osteoblasts dramatically reduced osteoblast differentiation ability, and MACF1-KD cells do not respond to MU conditions. Mechanistically, MACF1 may mediate the inhibitory effect of MU on osteoblast differentiation via the F-actin/ERK/Runx2 signal axis, demonstrating MACF1 as a mechanotransduction mediator. This study contributes to a novel mechanistic insight of MU inhibiting osteoblast differentiation and cell mechanotransduction.

MACF1, a widely expressed cytoskeletal protein, plays important roles in regulating cell functions, such as cell migration, proliferation, and differentiation [17,23,25,34,35]. Our previous studies have shown that MACF1 promotes osteoblast differentiation [24,25]. Moreover, studies have shown that MACF1 regulates cardiomyocyte adaptation to hemodynamic overload [36], indicating a role of MACF1 in responding to mechanical stimuli. However, whether MACF1 responds to MU and participates in the decreased osteoblast differentiation induced by MU is unknown. In this study, MACF1 expression was decreased together with the reduced osteoblast differentiation under MU conditions. These findings demonstrate the response of MACF1 to MU and suggest that MACF1 may play a key role in MU inhibiting osteoblast differentiation.

To further confirm the role of MACF1 in MU inhibiting osteoblast differentiation, stable MACF1-KD MC3T3-E1 osteoblasts and MACF1-KD primary osteoblasts were adopted and treated by the MU condition. We found that MU significantly decreased the MACF1 expression and inhibited osteoblast differentiation in control cells (MACF1-CON). It is noteworthy that the osteoblast differentiation capacity of MACF1-KD/si-MACF1 cells under control conditions was similar to that of MACF1-CON cells under MU, and MU treatment did not cause any additional changes in osteoblast differentiation in MACF1-KD/si-MACF1 cells. In classical genetics and signaling analysis, this lack of an additive or synergistic effect establishes a definitive epistatic relationship (Genetic Epistasis) between MU and MACF1, suggesting that MACF1 is a major downstream mediator of MU treatment in osteoblasts.

As MACF1 is a critical cytoskeletal protein linking both F-actin and microtubules [16,37], which are regarded as important force-sensing units in cells [38], we speculated that MACF1 may transduce the MU signal via the cytoskeleton. Moreover, evidence shows that modulation of F-actin cytoskeleton influences signaling pathways involved in cell differentiation [39,40]. Thus, we focused on F-actin. The F-actin staining results showed that MU disrupted the normal distribution of F-actin in control (MACF1-CON) osteoblasts. Similar results were observed in MACF1-KD osteoblasts compared to the MACF1-CON cells. But interestingly, MU did not induce further alterations of F-actin distribution in MACF1-KD cells. These findings suggest that MACF1 is important in mediating the regulatory role of MU in F-actin distribution. To confirm whether the altered distribution of F-actin affects osteoblast differentiation, Cyto B was adopted to interrupt the F-actin distribution to detect the effects on osteoblast differentiation. The results showed that Cyto B induced the altered distribution of F-actin. Further, Cyto B treatment significantly decreased the ALP activity and downregulated the expression of osteogenic genes, including *ALP*, *Runx2*, *ColIα1*, and *Osterix*, demonstrating the inhibition effect of altered F-actin distribution on osteoblast differentiation. Several studies also show that the disruption of F-actin organization regulates osteoblast differentiation [41,42]. All these findings reveal MACF1-associated F-actin organization as a potential mediator of MU-induced impairment of osteoblast differentiation.

F-actin has been shown to link ERK and activate ERK signaling, which plays a key role in regulating osteoblast differentiation by regulating Runx2 [31,32,33,43]. Therefore, based on the above findings, we wondered if MACF1 mediates MU inhibiting osteoblast differentiation via the F-actin/ERK/Runx2 signal axis. To test this notion, we detected the expression and activation of ERK (phosphorylated ERK, p-ERK) in both MACF1-CON and MACF1-KD cells treated by the MU condition. The results showed that MU dramatically decreased p-ERK levels in MACF1-CON cells while there was no significant alteration of p-ERK in MACF1-KD cells either treated or non-treated by the MU condition. In classical genetic and pathway analysis, this lack of an additive effect in the KD background provides strong evidence of epistasis. Because MU has no effect on ERK phosphorylation when MACF1 is missing, it logically suggests the essential role of MACF1 in mediating mechanical signal transduction. Fassett et al. have reported that MACF1 is important for cardiomyocytes adapting to hemodynamic overload [36]. All of this demonstrates MACF1 as a mechanotransduction mediator. Similar results were observed for the expression of Runx2, downstream of ERK signaling and a key transcription factor for osteoblast differentiation [44,45], presenting as no significant changes in total Runx2 level between MACF1-KD cells and MACF1-KD cells treated by MU. This suggests the key role of MACF1 in regulating ERK/Runx2 signaling. Due to the phosphorylation effect of ERK on Runx2, the p-Runx2 levels were also detected in both MACF1-CON and MACF1-KD cells under MU conditions. Interestingly, both MU and MACF1-KD increased the p-Runx2 (Ser340) level of osteoblasts, while there was no significant alteration of p-Runx2 (Ser340) levels in MACF1-KD cells either treated or nontreated by the MU condition. As studies reveal that activation of ERK (p-ERK) induces p-Runx2 and mainly phosphorylates Runx2 at Ser301 and Ser319 [43,46,47], our findings are different from the previous studies. Because there are many sites in Runx2 to be phosphorylated, the different findings may be due to the different phosphorylation sites. Kugimiya et al. have reported that glycogen synthase kinase 3β (GSK3β) phosphorylates Runx2 at Ser369/Ser373/Ser377, which leads to Runx2 inactivation and proteasomal degradation, thereby suppressing osteoblast differentiation [48]. As ERK activation inhibits GSK3β activity [49,50], MACF1-KD suppresses ERK activity and our previous studies demonstrate that MACF1-KD increases the active level of GSK3β [24], these findings suggest the possibility that the increased p-Runx2 may be modulated by GSK3β.

To further confirm the involvement of the F-actin/ERK/Runx2 signal axis in MACF1 mediating MU inhibiting osteoblast differentiation, firstly, the alterations of ERK/Runx2 in osteoblasts treated by Cyto B were detected. The results showed that Cyto B treatment significantly reduced p-ERK level and total Runx2, but increased p-Runx2, demonstrating that ERK/Runx2 functions downstream of F-actin. Moreover, the ERK inhibitor PD98059 was adopted to inhibit ERK signaling activation to detect its effects on Runx2 expression and osteoblast differentiation in both MACF1-CON and MACF1-KD cells. PD98059 treatment significantly inhibited p-ERK levels in both MACF1-CON and MACF1-KD cells, demonstrating that ERK is downstream of MACF1. In addition, PD98059 treatment dramatically inhibited total Runx2 level but increased p-Runx2 level, which is similar to the results obtained from MU treatment. Furthermore, PD98059 treatment significantly inhibited osteoblast differentiation in both MACF1-CON and MACF1-KD cells. Interestingly, PD98059 treatment causes an additional decrease in osteoblast differentiation in MACF1-KD cells, which is not observed with MU. Based on the above findings of this study, MACF1 plays a key role in osteoblasts responding to MU and ERK functions downstream of MACF1. Therefore, MACF1-KD osteoblasts do not respond to MU, which does not cause further decreases in ERK activation and osteoblast differentiation. For PD98059 treatment, as PD98059 is an inhibitor of ERK and ERK functions downstream of MACF1, PD98059 directly inhibits ERK activation regardless of whether there is MACF1 or not. Thus, the further decrease in osteoblast differentiation with PD98059 treatment in MACF1-KD cells may be due to the higher inhibitory level of PD98059 on ERK activation compared to MU. All these findings reveal that MACF1 plays a key role in mediating the inhibitory effect of MU on osteoblast differentiation via the F-actin/ERK/Runx2 signal axis.

This study reveals MACF1 as a mechanotransduction mediator that participates in MU suppressing osteoblast differentiation and contributes to a novel mechanistic insight for osteoblast mechanotransduction. However, certain limitations persist and require further research. The present study mainly adopted the stable MACF1-KD MC3T3-E1 osteoblast cell line without conducting MACF1 overexpression experiments. The stable MACF1-KD osteoblast cell line, established via highly specific lentivirus-mediated shRNA, serves as a well-validated model in our laboratory [23,24,25]. Nevertheless, a rescue experiment via MACF1 overexpression will further confirm MACF1’s critical role in the inhibition of osteoblast differentiation induced by MU.

## 5. Conclusions

The present study investigates the role and mechanism of MACF1 in MU inhibiting osteoblast differentiation. The results show that MU conducted by a random positioning machine (RPM) inhibits osteoblast differentiation and downregulates MACF1 expression. Moreover, MACF1-KD reduces osteoblast differentiation ability and makes osteoblasts insensitive to MU, demonstrating the crucial role of MACF1 in mediating osteoblasts’ response to MU conditions. Further studies show the redistribution of F-actin and inactivation of ERK induced by both MU and MACF1-KD in control cells, but these are not significantly changed in MACF1-KD cells under MU compared to control conditions. Similar results are observed for Runx2, the key transcription factor for osteoblast differentiation and downstream effector of ERK signaling. All these findings demonstrate that MACF1 mediates the inhibitory effect of MU on osteoblast differentiation via the F-actin/ERK/Runx2 signal axis. This study indicates MACF1 as a mechanotransduction mediator in osteoblasts and contributes to a novel mechanistic insight for osteoblast mechanotransduction.

## Figures and Tables

**Table 1:** Primer sequences used for real-time PCR.

Gene Name	Sequences (5′–3′)
*MACF1*	F: 5′-GAAAACATTCACCAAGTGGGTCAAC-3′
	R: 5′-TGTCCATCCCGAAGGTCTTCATAG-3′
*ALP*	F: 5′-GTTGCCAAGCTGGGAAGAACAC-3′
	R: 5′-CCCACCCCGCTATTCCAAAC-3′
*Runx2*	F: 5′-TGCACCTACCAGCCTCACCATAC-3′
	R: 5′-GACAGCGACTTCATTCGACTTCC-3′
*Col1a1*	F: 5′-TGTTCGTGGTTCTCAGGGTAG-3′
	R: 5′- TTGTCGTAGCAGGGTTCTTTC-3′
*Osterix*	F: 5′-ATGCTCCGACCTCCTCAACTTT-3′
	R: 5′-GGCAAATAGGATTGGGAAGCA-3′
*GAPDH*	F: 5′-TGCACCACCAACTGCTTAG-3′
	R: 5′-GGATGCAGGGATGATGTTC-3′

## Data Availability

The original contributions presented in this study are included in the article/Supplementary Materials. Further inquiries can be directed to the corresponding authors.

## Supplementary Materials

The following supporting information can be downloaded at https://www.mdpi.com/article/10.3390/cells15161448/s1, Figure S1: MU inhibits MACF1 expression and osteoblast differentiation in primary osteoblasts. Figure S2: MACF1 KD inhibits osteoblast differentiation and is involved in the inhibition effect of MU on osteoblast differentiation detected in primary osteoblasts. Figure S3: Knockdown of MACF1 in primary osteoblasts alters F-actin distribution and MACF1 is involved in the inhibition effect of MU on osteoblast differentiation by regulating F-actin distribution.

## Abbreviations

The following abbreviations are used in this manuscript:

MACF1	Microtubule actin crosslinking factor 1
ERK	Extracellular signal-regulated kinase
Runx2	Runt-related transcription factor 2
MACF1-KD	MACF1 knockdown
RPM	Random positioning machine
ALP	Alkaline phosphatase
α-MEM	Alpha-minimum essential medium
FBS	Fetal bovine serum
shRNA	Short hairpin RNA
Cyto B	Cytochalasin B
BCIP	5-Bromo-4-chloro-3-indolyl phosphate
NBT	Nitro blue tetrazolium
PBS	Phosphate-buffered saline
cDNA	Complementary DNA
GAPDH	Glyceraldehyde-3-phosphate dehydrogenase
Ct	Threshold cycle
HRP	Horseradish peroxidase
TX	Triton X-100
BSA	Bovine serum albumin
DAPI	4′,6-diamidino-2-fenilindol
SD	Standard deviation
ColIα1	Type I collagen α1
MU	Mechanical unloading
