# Supplementary material for: MACF1 Mediates the Impairment of Mechanical Unloading on Osteoblast Differentiation via F-Actin/ERK/Runx2 Axis
Source: Cells. 2026 Aug 11;15(16):1448. doi: 10.3390/cells15161448 (PMC13510352; doi:10.3390/cells15161448)
Supplement: Supplementary file 1 [file cells-15-01448-s001.zip › cells-4323811-supplementary.pdf]

Supplementary methods

Primary osteoblasts isolation and cell culture

The primary osteoblasts were isolated from the calvaria of neonatal (1 – 3 day old) C57BL/6 mice. Briefly, the calvarium was isolated by surgery and then washed with phosphate-buffered saline (PBS) to remove the soft tissues and related connective tissue. Then, the cells were released by five sequential 10 min digestion with collagenase II (Sigma, Germany) and dispase (Invitrogen, USA). Cell populations from digestions 4 to 5 were collected by centrifugation and pooled. The obtained cells are primary murine osteoblasts. The primary osteoblasts were cultured in alpha minimum essential media (αMEM) containing fetal bovine serum (FBS), treptomycin and penicillin and at 37℃ with 5% CO_2, the cells were cultured in a humidified incubator. To knockdown the expression of MACF1 in primary osteoblasts, primary murine osteoblasts were transfected with small interfering RNA (siRNA) of MACF1, and its corresponding control siRNA (Tsingke Biotech, China) respectively by using Lipofectamine 2000 Reagent (Life Technologies, China). For the induction of osteoblast differentiation, cells were cultured with osteogenic medium which contained the culture medium supplemented with 50 μg/ml ascorbic acid (AA) (Sigma, USA) and 10mM β–glycerophosphate (β-GP) (Sigma, USA). For cytochalasin B (Cyto B) experiments, primary osteoblasts were treated with 1 μM Cyto B for 24h (hours).

Fluorescence staining

Fluorescent staining of the F-actin was conducted by using CoraLite® Plus 488-conjugated Phalloidin (Proteintech, China) according to the manufacturer's instruction. Briefly, cells were fixed with 4% paraformaldehyde for 20 min and rinsed for 5 min with TBS, 10 min with TBS-0.5% TritonX-100 (TX), and 3 × 5 min with TBS-0.1%TX, and then blocked with 2% bovine serum albumin (BSA) and 0.1% sodium azide in 0.1% Triton X-100 (TBS) for 10 min at room temperature. Then, the cells were incubated with Phalloidin at 4°C overnight. After overnight incubation, the cells were incubated with 5 μg/mL DAPI (4′,6-diamidino-2-fenilindol) (Beyotime, Shanghai, China) for 10 min to stain nucleus. Finally, the cells were enveloped with Fluoromount-G (Southern Biotech, USA) and observed by laser scanning confocal microscopy (Leica TCS SP8, Germany).

All other related experiment methods used for primary osteoblasts are the same as that used for MC3T3-E1 osteoblasts as described in “Methods”.

Supplementary Figures

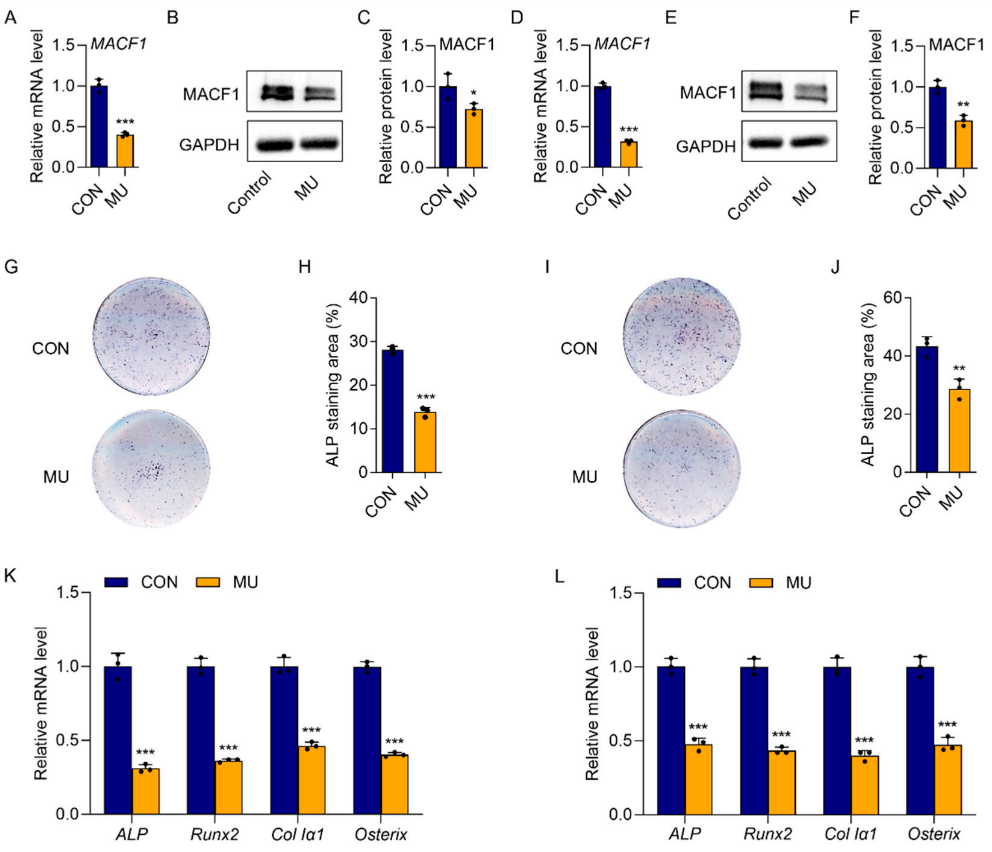

Figure S1. Mechanical Unloading (MU) inhibits MACF1 expression and osteoblast differentiation in primary osteoblasts. (A) The expression of MACF1 in primary murine osteoblasts was examined by real time PCR after 24h treatment of MU. (B and C) Representative western blots of MACF1 in primary osteoblasts treated by 24h MU (B) and the quantification results with GAPDH as internal control (C). (D) The expression of MACF1 in primary cells was examined by real time PCR after 48h treatment of MU. (E and F) Representative western blots of MACF1 in primary cells treated by 48h MU (E) and the quantification results with GAPDH as internal control (F). (G and H) ALP activity of primary osteoblasts was detected by ALP staining after 24h treatment of MU (G) and the quantification of ALP staining area (H). (I and J) ALP activity of primary cells was detected by ALP staining after 48h treatment of MU (I) and the quantification of ALP staining area (J). (K and L) The expression of ALP, Runx2, ColIα1 and Osterix

in primary cells examined by real time PCR after 24h (K) and 48h (L) treatment of MU respectively. CON: Normal control condition, MU: mechanical unloading. Data are presented as mean value ± SD and the Student's t-test was used for statistical analysis. **P*<0.05, ***P*<0.01, ****P*<0.001.

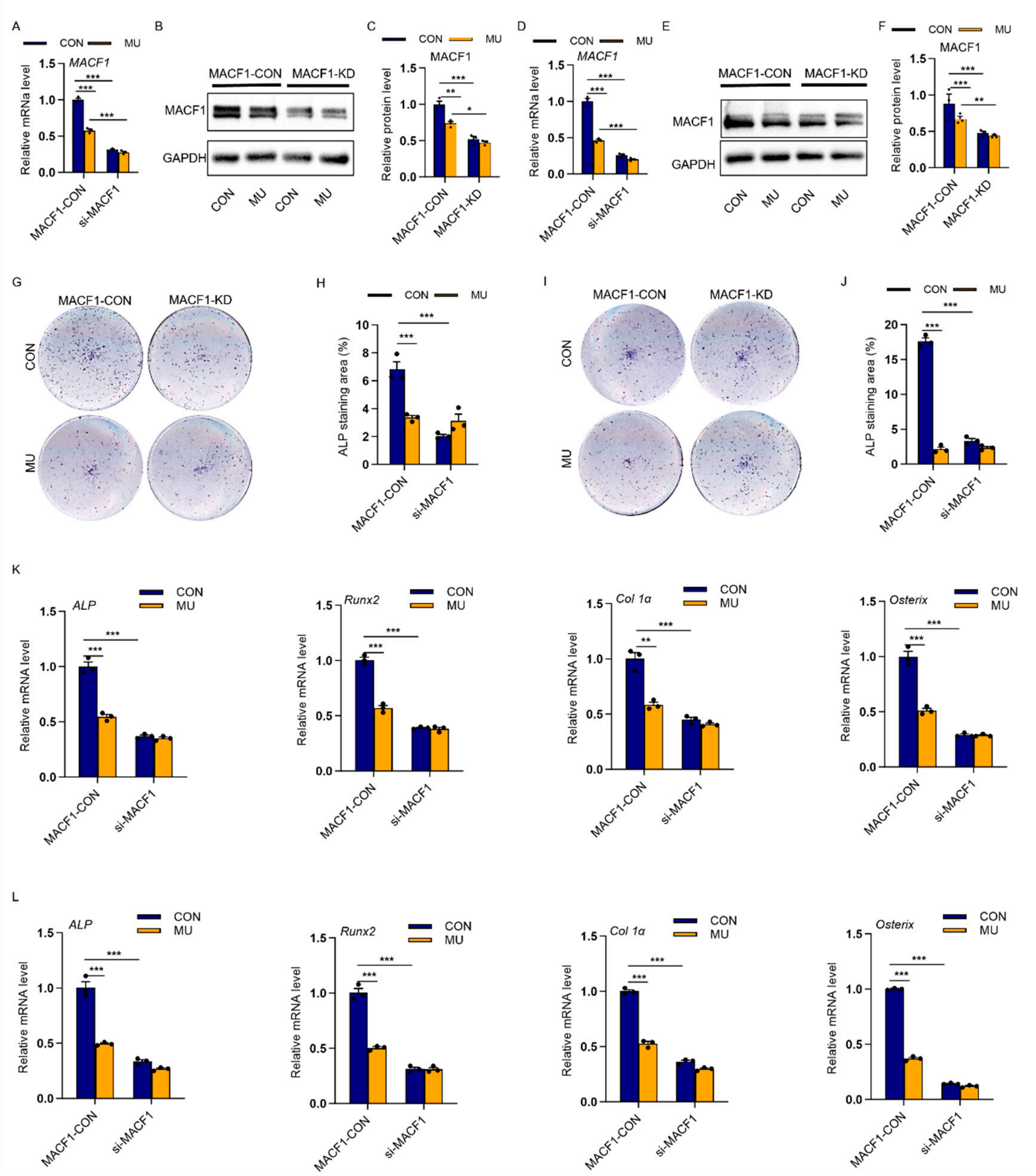

Figure S2. MACF1 knockdown inhibits osteoblast differentiation and mediates the inhibition effect of mechanical unloading on osteoblast differentiation detected in primary osteoblasts. (A) The expression of MACF1 examined by real time PCR after 24h treatment of MU in Control (MACF1-CON) and MACF1 knockdown (si-MACF1) primary osteoblasts. (B and C) Representative western blots of MACF1 treated by 24h MU in MACF1-CON and si-MACF1 cells (B) and the quantification results with GAPDH as

internal control (C). (D) The expression of MACF1 examined by real time PCR after 48h treatment of MU in Control (MACF1-CON) and si-MACF1 cells. (E and F) Representative western blots of MACF1 treated by 48h MU in MACF1-CON and si-MACF1 cells (E) and the quantification results with GAPDH as internal control (F). (G and H) ALP activity was detected by ALP staining after 24h treatment of MU in MACF1-CON and si-MACF1 cells (G) and the quantification of ALP staining area (H). (I and J) ALP activity was detected by ALP staining after 48h treatment of MU in MACF1-CON and si-MACF1 cells (I) and the quantification of ALP staining area (J). (K and L) The expression of ALP, Runx2, ColIα1 and Osterix examined by real time PCR after 24h (K) and 48h (L) treatment of MU in MACF1-CON and si-MACF1 cells, respectively. CON: Normal control condition, MU: mechanical unloading, MACF1-CON: Control primary osteoblasts, si-MACF1: MACF1 knockdown primary osteoblasts. Data are presented as mean value ± SD and the two-way ANOVA with post hoc Tukey's test was used statistical analysis. *$P<0.05$, **$P<0.01$, ***$P<0.001$.

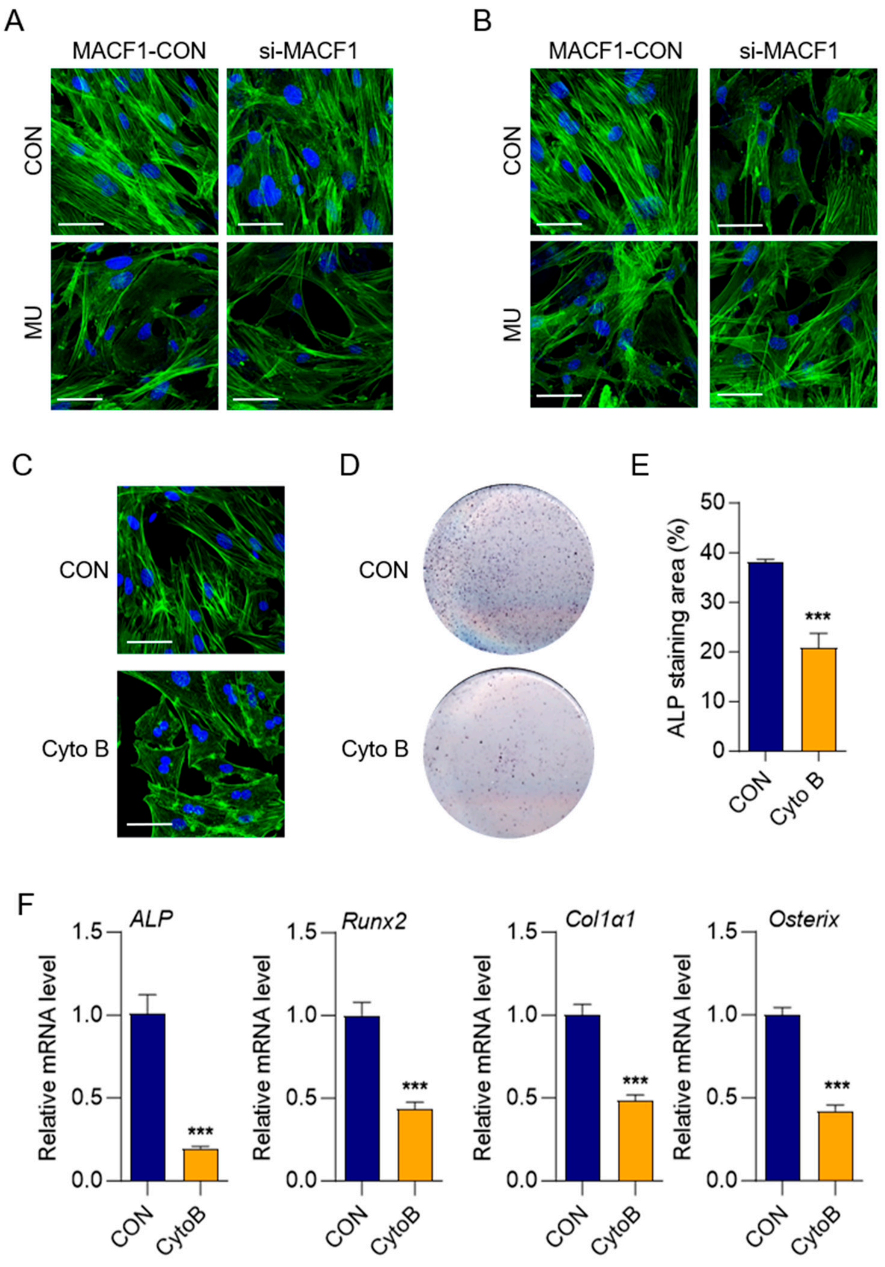

Figure S3. Knockdown of MACF1 in primary osteoblasts alters F-actin distribution and MACF1 mediates the inhibition effect of mechanical unloading on osteoblast differentiation by regulating F-actin distribution. (A) F-actin distribution was detected by fluorescence staining after 24h treatment of MU in MACF1-CON and MACF1 knockdown (si-MACF1) primary osteoblasts. Bar: 50μm. (B) F-actin distribution was detected by fluorescence staining after 48h treatment of MU in MACF1-CON and si-MACF1 cells. Bar: 50μm. (C) F-actin distribution in primary osteoblasts was detected by fluorescence staining after 1μM Cyto B treatment for 24h. Bar: 50μm. (D and E) ALP activity was detected by ALP

staining after 1μM Cyto B treatment for 24h (D) and the quantification of ALP staining area (E). (F) The expression of ALP, Runx2, CollIα1 and Osterix examined by real time PCR after 1μM Cyto B treatment for 24h. CON: Normal control condition, MU: mechanical unloading, MACF1-CON: Control primary osteoblasts, si-MACF1: MACF1 knockdown primary osteoblasts, Cyto B: cytochalasin B treatment. Data are presented as mean value ± SD and the Student's t-test was used for statistical analysis. ***P<0.001.
